# Pejotização and implications for nursing work in Brazil: repercussions of neoliberalism

**DOI:** 10.1590/1980-220X-REEUSP-2022-0396en

**Published:** 2023-05-26

**Authors:** Sheila Nascimento Pereira de Farias, Norma Valéria Dantas de Oliveira Souza, Thereza Christina Mó y Mó Loureiro Varella, Karla Biancha Silva de Andrade, Samira Silva Santos Soares, Eloá Carneiro Carvalho

**Affiliations:** 1 Universidade Federal do Rio de Janeiro, Escola de Enfermagem Anna Nery, Rio de Janeiro, RJ, Brazil.; 2 Universidade do Estado do Rio de Janeiro, Faculdade de Enfermagem, Rio de Janeiro, RJ, Brazil.

**Keywords:** Nursing, Occupational Health, Working Conditions, Work Hours, Enfermería, Salud Laboral, Condiciones de Trabajo, Horas de Trabajo, Enfermagem, Saúde Ocupacional, Condições de trabalho, Jornada de trabalho

## Abstract

**Objective:**

To analyze *pejotização* in the context of nursing work and the respective implications for the protection and health of these professionals.

**Method:**

Documentary study, which had news, resolutions, and recommendations issued by the Federal Nursing Council and the Regional Nursing Councils as data collection source, which underwent lexical analysis, based on data processing using the software Iramuteq.

**Results:**

Six news items were captured for analysis. The similitude analysis was built with 40 active forms and six discussion centers were generated, with the most expressive lexicons in each of these being outsourcing, economic, *pejotização*, deputy, Federal Nursing Council, Bill of Law.

**Conclusion:**

In the quest to increase capital based on neoliberal ideas, strategies are produced that put the workers’ and users’ health and safety at risk. *Pejotização* leads to loss of labor rights, as it deprives the worker of consolidated labor achievements, such as the 13th salary, paid vacations, sick leave and, above all, insecurities are generated in relation to the future, with few guarantees, causing negative impacts on these workers’ health.

## RESUMO

**Objetivo:**

Analisar a pejotização no contexto do trabalho de enfermagem e as respectivas implicações para proteção e saúde desses profissionais.

**Método:**

estudo documental, que teve como fonte de coleta de dados notícias, resoluções e recomendações veiculadas pelo Conselho Federal de Enfermagem e pelos Conselhos Regionais de Enfermagem e submetidas à análise lexical, a partir do processamento dos dados pelo software Iramuteq.

**Resultados:**

Captaram-se seis notícias para análise. A análise de similitude foi construída com 40 formas ativas e geraram-se seis núcleos de discussão, sendo os léxicos mais expressivos em cada um destes: terceirização, econômico, pejotização, deputado, Conselho Federal de Enfermagem, Projeto de Lei.

**Conclusão:**

Na busca por incremento do capital no ideário neoliberal, produzem-se estratégias que colocam em risco a saúde e a segurança de trabalhadores e usuários. Existe perda de direitos trabalhistas, por meio da pejotização, negando-se ao trabalhador conquistas laborais consolidadas, como 13º salário, férias remuneradas, licença por adoecimento e, sobretudo, geram-se inseguranças em relação ao futuro, com poucas garantias, ocasionando impactos negativos na saúde desses trabalhadores.

## RESUMEN

**Objetivo:**

Analizar la *pejotização* en el contexto del trabajo de enfermería y las respectivas implicaciones para la protección y la salud de estos profesionales.

**Método:**

Estudio documental, que tuvo como fuente de recolección de datos noticias, resoluciones y recomendaciones emitidas por el Consejo Federal de Enfermería y los Consejos Regionales de Enfermería y sometidos a análisis del léxico, a partir del procesamiento de datos utilizando el software Iramuteq.

**Resultados:**

Se capturaron seis noticias para análisis. El análisis de similitud se construyó con 40 formas activas y se generaron seis núcleos de discusión, con los léxicos más expresivos en cada uno de estos siendo terceirización, económico, *pejotização*, diputado, Consejo Federal de Enfermería, Proyecto de Ley.

**Conclusión:**

En la búsqueda por aumentar el capital y las ideas neoliberales se producen estrategias que ponen en riesgo la salud y seguridad de los trabajadores y usuarios. Se pierden los derechos laborales, a través de la *pejotização*, negándose al trabajador conquistas laborales consolidadas, como el salario 13, vacaciones pagadas, bajas por enfermedad y, sobre todo, se generan inseguridades en relación al futuro, con pocas garantías, provocando impactos negativos. sobre la salud de estos trabajadores.

## INTRODUCTION

Neoliberalism in Brazil has been intensifying since 2015 and is associated with a narrative based on the defense of ­economic growth and development, as well as the increase in the number of job openings. However, the result observed of the radical neoliberal agenda implemented is the flexibilization of labor relations based on the reduction of workers’ rights, which was materialized, among other expedients, through the labor reform^(1)^, the increase in economic recession, and unemployment in the country.

In the neoliberal perspective, the State does not participate in employment relations, with the parties, employer and employee, having the responsibility of concluding employment contracts^(2)^. These measures are restrictive of rights and result from changes in legislation that redirect the action of the State and have as their main goal the protection of capital to the detriment of the Brazilian worker. Some of these measures are found in Law 13.467/2017, which modified approximately 201 articles of the Consolidation of Labor Laws (CLT – a decree which governs labor relations in Brazil), and in Law 13.429/2017^(3,4,5)^, expanding the possibilities of outsourcing and temporary contracts.

From this perspective, a gradual loss of social and labor rights has been observed, since these laws are infraconstitutional and oppose the principles of the 1988 Federal Constitution (CF), called the Citizen Constitution, which in art. 7 safeguards the fundamental rights of the Brazilian people.

It is worth analyzing that the turning point occurred in the deconstruction of social rights. These actions sustainability took place under three aspects: flexibility of the employment ­relationship; weakening of union power and public institutions; and individualization of occupational risks. There was an inversion of the State’s protective logic, bringing up the logic of entrepreneurship, which abandons the worker to his own fate, and of employment maintenance at any cost, to the detriment of protecting the worker and his salary^(1)^.

In light of the labor reform, Law no. 13.467/2017, the ­*pejotização/PJ* (employer´s requirement that service providers turn into legal entities to be hired as independent contractors) is an institute that presents itself by hiring a worker, individual or legal entity. That is, the employer hires the employee as a legal entity. This way, the legal bond is governed by Civil Law, decharacterizing the employment relationship^(2)^. In this logic, what has happened is that companies fire employees hired under the CLT regime (the Consolidation of Labor Laws, a decree which governs labor relations in Brazil) and replace them with legal entities that are often represented by a single person. This can be considered a way to circumvent the worker’s protective legislation and employment relations.

In the health area, *pejotização* is widely used for the provision of medical professionals, justified by the high market wages for this professional category. However, currently, it has been used as an alternative for hiring other health professionals^(6)^, such as nurses, with the justification of hiring staff for a ‘temporary’ period, through the bidding of outsourced companies or even by hiring a legal entity (*pejotização*)^(7)^.

Therefore, it is observed that this new logic, in addition to violating the constitutional principle of no social regression, restricts workers’ rights guaranteed for decades by the Constitution, by the principles of the labor law and international treaties and conventions related to workers’ rights. In this context, this article aimed at analyzing the *pejotização* of nursing work and the respective implications for the protection and health of these professionals.

## METHOD

### Design and Field of Study

This is a documentary study, whose source of data collection was news, resolutions, and recommendations issued by the Federal Nursing Council (COFEn) and by the Regional Nursing Councils (COREn). Documentary studies allow in-depth analysis of raw information by selecting, treating and interpreting data dispersed in written materials, giving them due value as a source of research guided by an objective^(8)^.

### Data Collection

It took place in July 2022, on the Cofen and Coren websites, using a form with the following aspects as a way of collection: type of document; publication date of the document; author of the material; reason for elaboration; elements related to *pejotização*. The first stage of data collection consisted of cataloging the news and information containing the term “pejotização”, which was typed in the search box available on the sites mentioned. The second stage involved a detailed reading of the publications’ content and organization in a single file, so that it could later be organized in the format of a “textual corpus” to be processed by the lexical analysis software.

There was no filter with a temporal cut, in the expectation of identifying the largest amount of news involving the theme. As an inclusion criterion, the relationship with the theme was considered; as exclusion criteria, duplication of content.

Initially, 17 publications were found and, after reading the titles and viewing the complete material, eliminating duplicates, six publications remained in the corpus.

**Figure 1:**
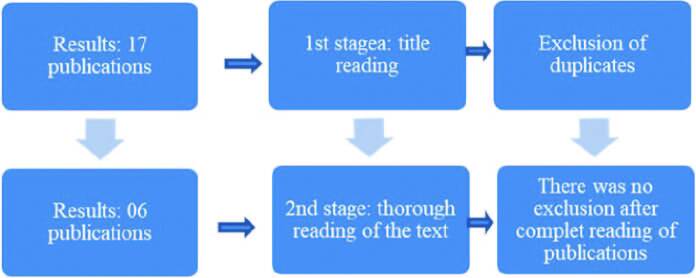
Corpus constitution process. Rio de Janeiro, RJ, Brazil, 2022.

This stage of data collection and organization was carried out by two authors. The synthesis of the methodological process of constitution of the corpus is illustrated in Figure 1.

It should also be noted that the selected publications had their electronic addresses identified and arranged in a Microsoft Excel 2016 spreadsheet. Thus, researchers could have unrestricted access to the material and consult whenever necessary.

### Data Analysis and Treatment

For data analysis, the Interface de R pour Analyzes Multidimensionnelles de Textes Et de Questionnaires (Iramuteq) software, version 7.2, was used, which has five possibilities for data processing: i) statistical (lexicographic) analysis, ii) specificity and factorial correspondence analysis, iii) descending hierarchical classification (DHC), iv) similitude analysis, and v) word cloud^(9)^.

For the purposes of this study, statistical analysis was initially chosen, since this resource allows identifying the frequency of the lexicon in the corpus, that is, the words that appear in certain contexts and that can be investigated based on the recovery of the Text Segments (TS) in which these lexicons appear, through the “concordance” feature. Thus, this information was accessed to ratify the results of the graphical representations generated a posteriori by the analysis of similitude and word cloud.

Based on the similitude analysis, the existing link among the words in the corpus was observed. This type of analysis is based on graph theory, which allows identifying co-occurrences between words, whose results help the researcher to identify the structure of a textual corpus^(10)^. From this analysis and from the graphic representation, the font size of the words, the thickness of the lines that connect them and the polygon to which they belong can be observed.

In its turn, the word cloud graphically represents the words according to the frequency of appearance in the texts. Thus, the larger words were those that appeared more often in the text, and the smaller ones appeared less frequently^(9,10)^.

The selection of these analysis techniques took place due to the fact that they allow the researcher to organize the vocabulary in a simple, understandable, and focused way^(10)^. In addition, from an analytical point of view, they allow the integration of quantitative and qualitative analysis, with a view to minimizing subjectivity bias and allowing advances in data interpretation.

Thus, based on the most prevalent lexicons in the similitude analysis and in the word cloud, and the TSs in which they appeared, the resulting themes that could be discussed were identified based on the theoretical framework.

### Ethical Aspects

There was no need to submit this study to an ethics committee, as we worked with data that were public. However, other aspects of research ethics were revealed, as recommended by Resolution 466/2012.

## RESULTS

**Chart 1:**
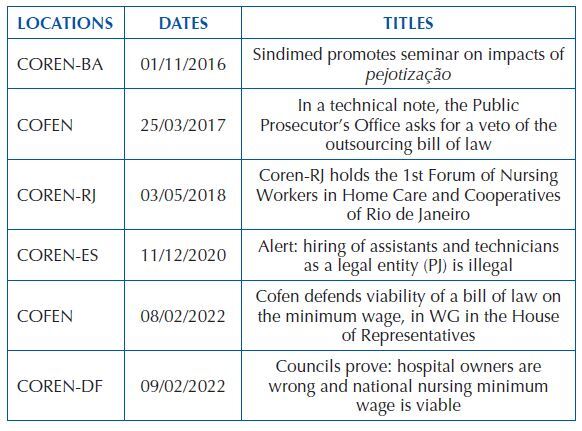
Exposure of the place, date and title of the documents on *pejotização*. Rio de Janeiro, RJ, Brazil, 2022.

Six pieces of news were captured for analysis, which were published between the years 2016 and 2022. It should be noted that two of these pieces of news were published by Cofen, and the other four were shared by the following sections: Coren-BA, Coren-RJ, Coren-ES, and Coren-DF. Chart 1 presents the location, date and title of the selected news. Furthermore, it was found that in 11 sections none of the news dealt with the topic researched and another 11 sections of Coren republished only the Cofen note in defense of the minimum wage (which encompasses the researched discussion).

**Figure 2:**
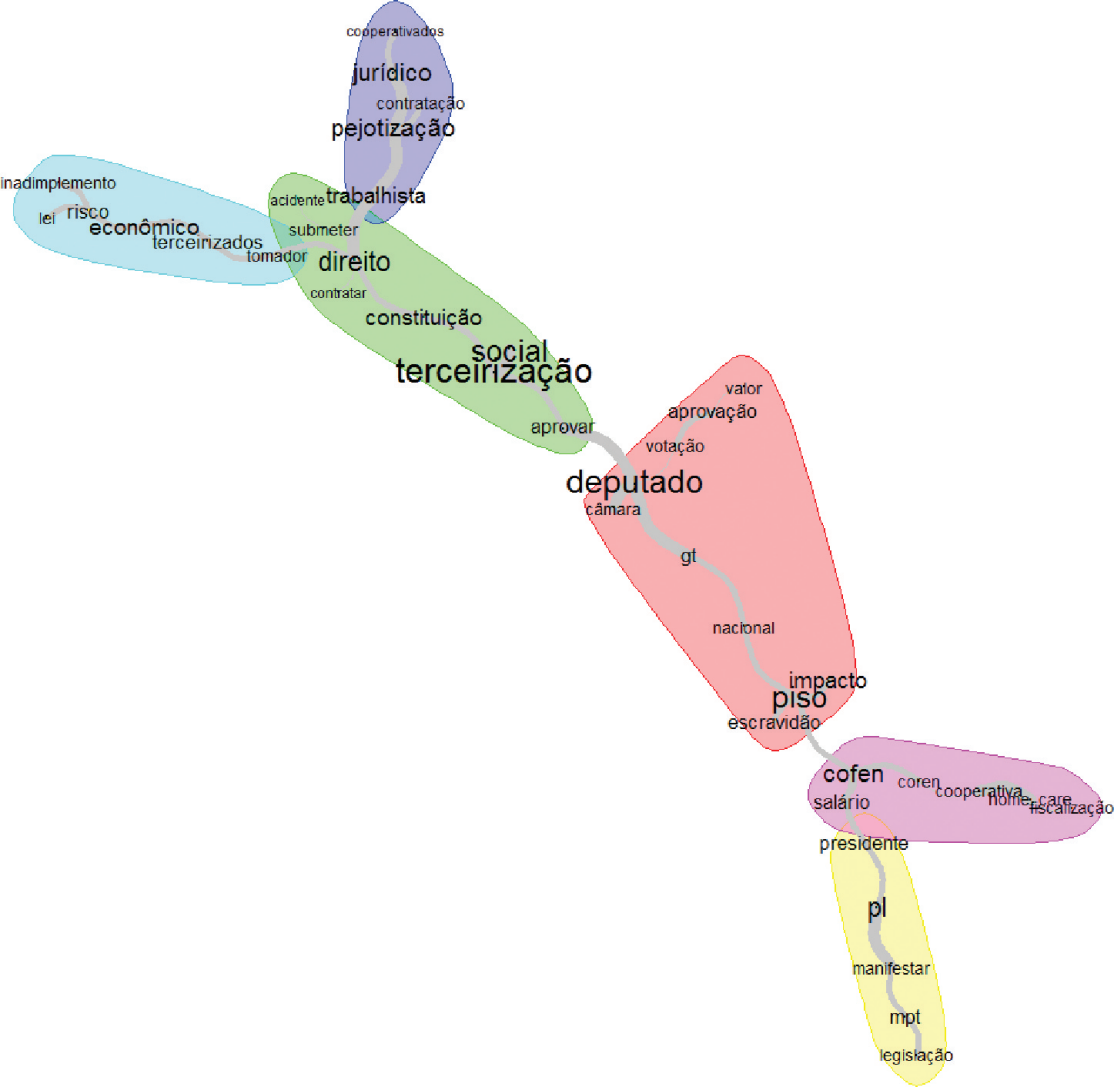
Similitude Analysis. Rio de Janeiro, RJ, Brazil, 2022.

Source: Research data, 2022.

The similitude analysis was built with 40 active forms and six discussion centers were generated, with the most ­expressive lexicons in each of these being outsourcing (*terceirização)*, economic (*econômico*), *pejotização*, deputy (*deputado*), Federal Nursing Council, Bill of Law (*projeto de lei*). Figure 2 allows observing the tree of words with the branches based on the relationships kept between the words in the texts.

**Figure 3:**
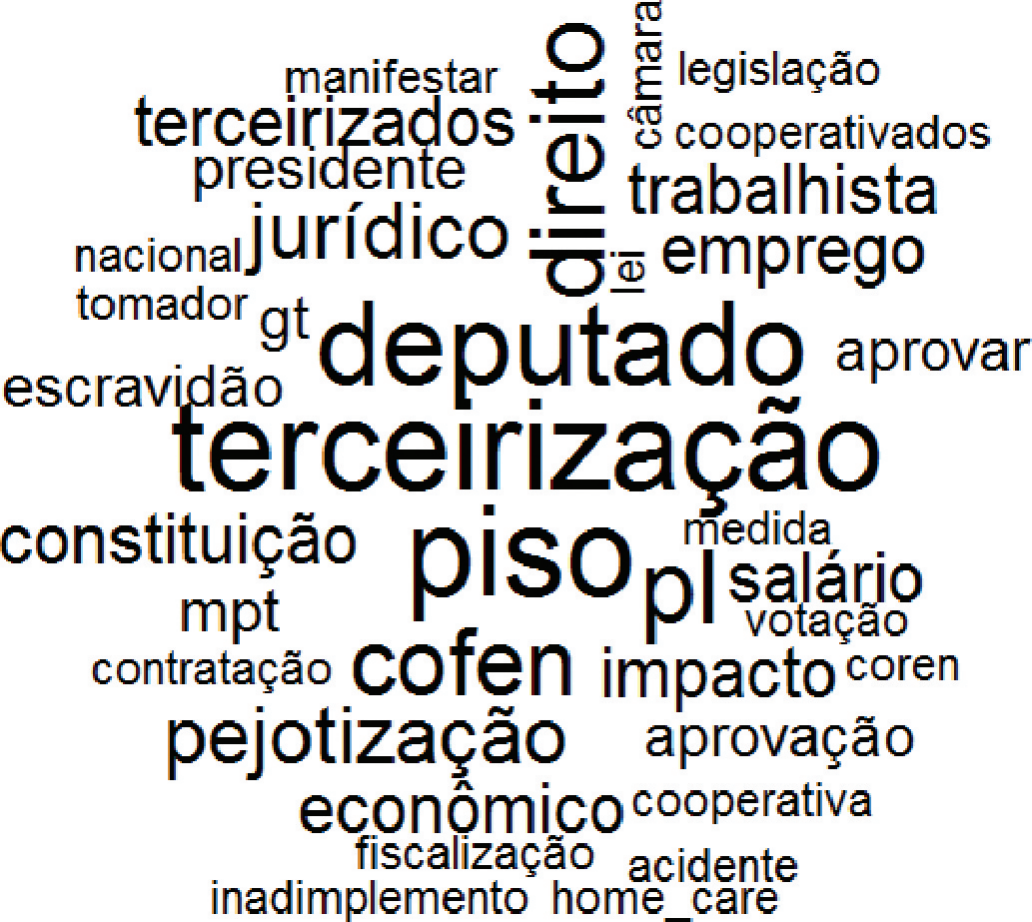
Word cloud. Rio de Janeiro, RJ, Brazil, 2022.

Source: Research data, 2022.

Figure 3, resulting from the word cloud, organized the words according to the frequency they appeared in the corpus. Thus, the words “outsourcing” (*terceirização*) and “minimum wage” (*piso*) are highlighted, as they appeared in the text 10 times; then the words deputy (*deputado*) (9x), right (*direito*) (8x), Cofen (7x) and *pejotização* (7x).

From an analytical point of view, when recovering the Text Segments, which deal with outsourcing, the following stand out:

*The **outsourcing** of services constitutes a practice that deeply weakens the effectiveness of workers’ fundamental rights provided for in art. 7 of the constitution, in view of the deleterious repercussions it entails on working conditions (p. of news 1).*

*This practice reduces human labor to the condition of merchandise and subverts the logical meaning of **outsourcing** which, according to management science, resides in the subcontracting of ancillary activities, back-office activity, to allow the contracting company to focus on its main activity, the target activity (p. of news 1).*

*the effect of unrestricted **outsourcing** is the mere replacement of direct jobs with a better level of social protection for precarious job vacancies, as already observed in other countries that have adopted similar practices (p. of news 1).*

*Furthermore, the expansion of **outsourcing** tends to increase the number and severity of accidents, burdening social security which already bears the annual cost of more than 18 billion reais only with accidents involving formal workers (p. of news 1).*

Among the TSs that deal with the economic effects, the need to analyze the feasibility of the Bill of Law (PL) that deals with the nursing minimum wage and the caveat made by Cofen that nursing technicians and assistants cannot be considered individual entrepreneurs:

*At the second meeting of the WG [Working Group] created by the House of Representatives to analyze the **economic** feasibility of the national nursing minimum wage, cofen and coren presented data and information that prove it (p. of news 6).*

*They do not assume the technical and **economic** risks of the enterprise, which is why they cannot be considered individual entrepreneurs, it is also worth remembering that assistants and technicians can only work in nursing under the supervision of a nurse; this is provided for in Law 7498 86 for professional practice (p. of news 5).*

As for the TSs where the lexicon “deputy” appears, the joint action of Cofen/Coren and the nursing political representation in the House of Representatives is identified:

***Deputies** and entities compared the degrading wages of the category to slavery and defended that the resources to pay with dignity exist, it is an investment in health, not an expense, cofen was present with the advisors (p. of news 2).*

*The unanimous approval in the senate was the result of bridge building efforts with all involved (not 2).*

As for the TSs where the lexicons “COFEN” and “PL” appear, the following stand out:

*In the assessment of the president of Cofen, the current text of the **PL** 2564 is the result of a lot of talks and a pact and already takes budget limitations into account* (p. of news 2).

In addition, regarding the central theme of this article, when recovering the STs, in which the lexicon “pejotização” appears, the following are identified:

The so-called **pejotização** of nursing workers refers to hiring without an employment relationship or labor rights. Slave labor is being installed in the profession (p. of news 2).

*By authorizing the subcontracting of services in chain, the “quarteirização”, and favoring the hiring of subordinate workers as legal entities, the so-called **pejotização,** the bill of law contributes to the extreme precariousness of working conditions (p. of news 1)*.

*Companies have hired nursing technicians and assistants as legal entities, the already known **pejotização;** the maneuver, however, is considered labor fraud and can penalize both the employer and the contractor (p. of news 5)*.

## DISCUSSION

### Pejotização in Nursing Work and the Precariousness of the Employment Relationship

Contemporaneity brings a movement to adjust work rules and employment relationships around the world^(11)^. In this regard, globalization advocates the restructuring of production in the neoliberal system, in which the primacy is in the financial market, to the detriment of production, that is, in profit-seeking, a term that designates that the market economy allows the privileged ones to extract most of income, through capital accumulation, spoliation of the worker, protection in competition, either through monopoly, subsidy, or market reserve. Thus, there is pressure for changes in worker protection mechanisms and in the forms of hiring the workforce, as well as in the labor legislation in force around the world.

In the Brazilian context, the precariousness of nursing work is driven and becomes legitimate based on the political and administrative reforms taking place through partnerships with public and private capital, which is driven by hiring through outsourced and precarious contracts. Nurses live with the loss of labor and social security rights on a daily basis^(12)^, being subjected to unworthy working conditions imposed by the exploitation of workers by capital. This is a major social setback, which imposes great vulnerability on nurses, causing insecurity, illness, job readaptation, disability retirement, among others.

The neoliberal project established a set of laws in Brazil that proposed breaking the inclusive logic of labor law^(13,14)^. Law 13.467/2017, called labor reform, which brings in its core the flexibilization of labor contract relations, provided for the deconstruction of the infra-constitutional precepts for workers protection and safety, meeting the policies of neoliberalism and consolidating itself through the reduction of labor rights. This approval lead to loss of employees’ achievements, as well as attacks on the principles of labor law, consolidated in the Federal Constitution of 1988, proving to be a major setback.

From this perspective, labor relations become increasingly multiform in terms of legal possibilities and other forms of contracting with more flexible outlines have emerged, the so-called “new” or “atypical” forms of work, such as temporary work, part-time work, outsourcing, cooperative members, internships, false self-employed, contracts as a legal entity, which is *pejotização* ^(14)^.

The forms of hiring mentioned above were created as an option for traditional job hiring, with the aim of meeting market demands due to productive restructuring. With the economic crisis and growing unemployment, they start to acquire relevance as a model of occupation and paid work. These are usually contracts that cause changes in the duration of the relationship, whether daily, weekly or monthly, and in the degree of subordination between employees and employers^(14,15)^.

It is asserted that the strategy used to justify the preference for these flexible contracts is the protection of capital, which removes the focus from the centrality of the human being and the dignity of the human person, distorting employment relations, “making them more flexible”, claiming to create more job vacancies^(14,15)^.

In this sense, the pejotização of work stands out, in which a contract for the provision of services of a civil nature is carried out for the execution of activities, with this type of contract being then regulated by Civil Law and not by Labor Law. In this perspective, they are criticized by the courts and considered, by jurisprudence in Brazil, as a way of defrauding employment contracts and employment relationships^(14,15)^.

In Brazil, the study of working conditions and the respective impacts on nursing workers is a topic frequently addressed in scientific articles^(16)^, demonstrating that these workers suffer from precarious employment relationships and unsatisfactory working conditions. These precarious conditions result in insecurity and work overload, excessive physical and mental effort, double working shift. In this respect, there is the occurrence of physical and mental illnesses, stress, great dissatisfaction and desire to leave the profession. It is asserted that, for nursing, the context is paradoxical: while the world of work demands from this professional the constant scientific^(12)^ improvement, as well as of skills and handling of new technologies, working conditions deteriorate sharply.

This way, the insertion of *pejotização* in the context of nursing work relations has led to an increase in the exploitation of the workforce, granting the employer greater freedom to dismiss without penalties, reduce hours or resort to more hours of work, pay real wages that are lower than what the work parity requires, in addition to subdividing the working day, changing the schedules and characteristics of work activities^(12)^.

### Implications of Pejotização on the Nursing Workers’ Health

The labor reform resulted in the precariousness of work, engendered by outsourcing, deepening the forms of worker exploitation in different labor scenarios, including the expansion of exploitation in health and education target activities^(11)^.

In Brazil and France, the reforms generated a cut that transformed the labor market into a laboratory, with consequences that need to be studied from the worker’s perspective. However, it is clear that this context has resulted in a decrease in worker protection, generating an increase in instability that, in its turn, leaves workers at the mercy of companies, which create almost unattainable productivity targets, but which employees submit to for fear of unemployment and other negative repercussions for their life and material survival^(17-19)^.

As a form of even greater worker exploitation, neoliberal capital devises new tricks. It should be noted that *pejotização* reflects negatively on workers’ health due to precariousness and lack of safety and protection for workers. It affects the individual and human’s dignity. It masks the employment relationship, without the constitutional protection of the worker, causing an imbalance in contractual rules^(11,20)^.

In the health area, especially in nursing, the negative impacts caused by neoliberal ideas, which undoubtedly resulted in greater precariousness at work, and above all, the use of pejotização as a way of hiring workers, alludes to the increase in illness, presenteeism and absenteeism of these professionals who, in their turn, generate negative repercussions in the assistance to users of the health system^(15-21)^.

In the context of the coronavirus pandemic, all systems in the world were put to the test, as well as health workers, with different responses from different systems. In France, the health system was adjusted; however, there is still a call for relevant and updated policy measures to be adopted, so that researchers and policy makers can deal with the work to be done^(18)^. It is necessary to invest in information and better working conditions for health professionals to deal with the different periods: pandemic, pre-pandemic and post-pandemic.

Inadequate working conditions cause psychosocial and physical risks due to greater exposure to occupational hazards, which can worsen during pandemic periods. This context can translate into stress, depression, difficulty of adaption, insomnia, anxiety, burnout, panic syndrome, post-traumatic stress, and may even influence nurses, increasing the desire to abandon the profession^(22,23)^.

Thus, the reduction of the nursing workforce due to illness or evasion, due to the precariousness and inadequacy of working conditions, is serious for health systems, as it makes quality care unfeasible. In this perspective, investment has to be made in better working conditions and in the quality of work relationships, in organizational support, and in nursing leadership, as well as in the support to self-control and effectiveness of nursing professionals, through adequate training to provide care to the users of the health system^(17,21)^.

It is understood that the health sector presents specificities in the work process, such as dealing with human lives in atypical situations, such as the Covid-19 pandemic, which demands that the organization of work promote means for the work to develop with efficacy and efficiency. Moreover, in this context, it is necessary to strengthen the workforce, providing objective and subjective conditions, so that work activities occur properly^(11)^.

Thus, work precariousness, currently configured by the *pejotização* of nursing, is certainly not a means that will ensure quality in care, as it has the potential to make the worker sick due to the insecurity that this means of hiring promotes and due to the loss of labor rights. The perspective is that nursing professionals progressively present more illnesses, increased presenteeism and absenteeism or abandonment of the profession.

### Recent Developments on the National Nursing Minimum Salary

The struggle for the regulation of a national nursing minimum salary has, for decades, led to the need for this confrontation with capital holders. However, it should be noted that, currently, although there are advances for nursing, such as the recently approved Law 14.434/2022, which establishes the minimum wage for nursing at the national level, it is still necessary to generate efforts to guarantee its effective implementation, since precariousness still continues to permeate the nursing work context^(21)^.

With regard to the achievement of the minimum wage for nursing professionals, this acquisition is also a threat to the category, as there are rumors that there will be mass layoffs and a significant reduction in subsidies that guarantee working conditions. The perspective of this unfavorable scenario for nursing is based on the justification of the business community that there is no capital, that is, sustained profitability to pay the wage bill that has increased. Thus, despite the achievement, there are signs of more insecurities in the workforce.

By corroborating the above, in a recent event, for ­example, Minister Luis Roberto Barroso suspended the law approved by the National Congress, based on the argument that it can generate risks to employability and the quality of services in the health sector. This action of unconstitutionality was filed by the National Health Confederation (CNSaúde), which serves the interests of private health^(24)^.

It should be highlighted that, on the same day that the law was suspended, a note of grievance was published, signed by various entities, such as the Brazilian Association of Diagnostic Medicine (ABRAMED), the Brazilian Association of Health Plans (ABRAMGE), the Brazilian Association of Vaccine Clinics (ABCVAC), Brazilian Association of Dialysis and Transplant Centers (ABCDT), National Association of Private Hospitals (ANAHP), National Confederation of Municipalities (CNM), National Health Confederation (CNSAUDE), Confederation of Santas Casas and Philanthropic Hospitals (CMB), National Federation of Supplementary Health (FENASAUDE) and the Brazilian Federation of Hospitals (FBH)^(25)^.

In response, the minister suspended the Bill of Law 2.564/2020, based on the absence of forecasting the origin of the resources, which could overly burden public and private entities. And he gave a period of 60 days for public and private entities to comment on the financial impact^(26)^.

If, on the one hand, the approval of the law generated a lot of celebration on the part of the benefited class, on the other hand it is notorious that public and private entities pointed out concerns regarding the law. For example, the Ministry of Health argued that the impact of the increase would be BRL 22.5 billion as of 2021, reaching almost BRL 25 billion as of 2024. In addition, in relation to the private sector, a study carried out by the House of Representatives pointed to an impact of BRL 10.5 billion for the private hospital sector^(25)^.

However, COFEN refuted the argument used, stating that it had presented all the necessary studies and had a broad discussion of the financial impacts of the minimum wage during the course of the project^(25)^. In response to what happened, on 09/04/2022, COFEN published a note repudiating Minister Barroso’s preliminary decision and stating that “all budget impact studies were duly presented and discussed with all entities of the Union, States and Municipalities, in a plural and transparent manner before the National Congress”^(27)^. Moreover, he stressed that this decision “meets the pure convenience of the business class, who do not want to pay fair prices for the services provided by Nursing”^(27)^.

In this context, it is observed that, once again, neoliberal appeals weighed more than the importance and work of nursing, contributing to the precariousness of the category. The phenomenon of precariousness is complex and multifactorial. Therefore, the strengthening of class bodies, unions and associations is required to transform the adversities generated by this growing precariousness. The several entities linked to nursing need to act in defense of labor rights^(28–30)^. Also, the need for political representation of the category and mobilization of all nursing professionals is highlighted.

The contribution of this study is to discuss themes that are still little socialized in the health sector and, above all, in nursing, which are characterized as labor reform, *pejotização*, and impacts of neoliberalism on work in health and nursing. Furthermore, the discussion made in this article helps professionals to expand their critical capacity regarding the impacts of neoliberal ideas on workers’ health and safety, as well as get more prepared to develop strategies to minimize the repercussions of this context in their daily work.

The limitation of the study was the restricted obtainment of six documents to develop the analysis. A larger amount of news would contribute to a more comprehensive and therefore even more robust analysis.

## CONCLUSION

The neoliberal ideology, which has been strongly imposing itself in the health sector, is considered a misfortune and a disaster for both users and health workers, since human lives should not be seen as commodities that need to generate productivity and profit. In this quest to increase capital, strategies are engendered that endanger the health and safety of the social actors that permeate this context.

From this angle, loss of labor rights can be observed, through this new form of hiring personnel – *pejotização* – which deprives the worker of consolidated labor achievements, such as the 13th salary, paid vacations, sick leave and, above all, insecurities about the future, with little or no guarantees, are observed. Therefore, workers are denied the right to plan their lives, achieve better material conditions and quality of life.

This adverse work context generates negative impacts on workers’ health, with the potential for psychosomatic illnesses, premature retirement, and dropout from the profession. It also leads to repercussions for the quality of care, also promoting insecurity and risks for service users.
